# 

**Published:** 1887-09

**Authors:** 


### BRISTOL MEDICO-CHIRURGICAL JOURNAL

A Quarterly Journal of the Medical Sciences for the West of England and South Wales

*PUBLISHED UNDER THE AUSPICES OF*

*THE BRISTOL MEDICO-CHIRURGICAL SOCIETY.*

EDITOR:

J. GREIG SMITH, M.A., F.R.S.E.

ASSISTANT-EDITOR:

L. M. GRIFFITHS.

"Scire est nescire, nisi id me
Scire alius sciret."

BRISTOL: J. W. ARROWSMITH;

LONDON: J. & A. CHURCHILL, 11 NEW BURLINGTON STREET.

